# Non-linear association between serum spermidine and mild cognitive impairment: Results from a cross-sectional and longitudinal study

**DOI:** 10.3389/fnagi.2022.924984

**Published:** 2022-08-02

**Authors:** Jiahui Xu, Zhaoqing Sun, Rui Zhang, Ruixue Li, Zhecong Yu, Qianlong Zhang, Yanan Ma, Fuguo Xing, Liqiang Zheng

**Affiliations:** ^1^School of Public Health, Shanghai Jiao Tong University School of Medicine, Shanghai, China; ^2^Department of Cardiology, Shengjing Hospital of China Medical University, Shenyang, China; ^3^College of Public Health, Shanghai University of Medicine and Health Sciences, Shanghai, China; ^4^School of Public Health, China Medical University, Shenyang, China; ^5^Department of Clinical Epidemiology, Shengjing Hospital of China Medical University, Shenyang, China; ^6^Ministry of Education-Shanghai Key Laboratory of Children’s Environmental Health, Xinhua Hospital, Shanghai Jiao Tong University School of Medicine, Shanghai, China; ^7^Department of Biostatistics and Epidemiology, School of Public Health, China Medical University, Shenyang, China; ^8^Institute of Health Sciences, China Medical University, Shenyang, China; ^9^Institute of Food Science and Technology, Chinese Academy of Agricultural Sciences, Beijing, China

**Keywords:** spermidine, cognition, mild cognitive impairment, longitudinal study, rural

## Abstract

**Background:**

Although animal studies show that spermidine (SPD) affects cognitive function, the relevant evidence among humans is limited. We aim to examine the association between serum SPD levels and cognitive performance.

**Materials and Methods:**

We conducted a cross-sectional and longitudinal study including a baseline and one follow-up survey. The baseline survey was conducted from June 2019 to August 2019, while the follow-up survey was conducted from June 2021 to August 2021. We analyzed 3,774 adult participants aged >35 years, who had no history of dementia.

**Results:**

The mean (SD) age of the participants was 57.4 (9.8) years. Relative to the first tertile, the multivariate-adjusted ORs (95% CIs) of mild cognitive impairment (MCI) for the second and third tertile groups were 0.78 (0.65, 0.93) and 0.80 (0.67, 0.96), respectively. Restricted cubic spline models show that there is a non-linear association between SPD and MCI. In line with cross-sectional findings, the longitudinal study showed that a high SPD concentration may indicate a lower risk of MCI [ORs (95% CIs) for the third tertile of 0.62 (0.39, 0.99)].

**Conclusion:**

Our findings suggest that SPD is favorable for cognitive function. Monitoring the SPD levels may help reduce the incidence of MCI, hence decreasing the burden of MCI.

## Introduction

Dementia is the leading cause of disability among people aged over 65 years worldwide. From 1990 to 2016, the global number of dementia patients more than doubled, from 20.2 to 43.8 million (GBD 2016 Dementia Collaborators, 2019). People with dementia in China account for approximately 25% of the dementia patients (Jia et al., 2020) worldwide. This scenario has imposed a heavy socioeconomic burden, impacting their quality of life. Although China has established and implemented a series of programs to manage this disease in the past decade, patients in rural areas often have difficulty accessing health services compared with patients in urban areas (Xue et al., 2018). Meanwhile, no specific therapy is available for the treatment of dementia. Therefore, early identification of high-risk populations and improvement of prognostic factors are critical, especially for people living in rural areas.

Mild cognitive impairment (MCI), the stage between normal cognition (NC) and dementia, is associated with a higher risk of dementia. Previous studies have shown that approximately 16% of patients diagnosed with MCI return to normal or near-normal cognition after a year of treatment (Koepsell and Monsell, 2012). Thus, identifying more sensitive or specific biomarkers for MCI is essential to reduce the burden of dementia.

Spermidine (SPD) is a natural polyamine present in all living organisms, and play a key role in maintaining cellular homeostasis. This chemical contributes to various critical cellular functions, including cell growth and proliferation, tissue regeneration, DNA and RNA stabilization, enzymatic modulation, and regulation of translation (Madeo et al., 2019). The association between SPD and cognition has been explored using animal experiments. Gupta et al. (2013) demonstrated that fruit flies fed SPD showed enhanced autophagy and that SPD can effectively inhibit aging-associated memory impairment via autophagy. A recent study revealed that dietary SPD passes the blood–brain barrier and improves spatial and temporal memory in aged mice (Schroeder et al., 2021).

Several small randomized controlled clinical trials have been implemented to examine the relationship between dietary SPD supplements and cognition in humans; however, the conclusions have been inconsistent (Wirth et al., 2018; Pekar et al., 2021). Additionally, a prospective cohort study involving 815 participants found that higher nutritional SPD intake is associated with a lower risk for cognitive impairment and decline (Schroeder et al., 2021). However, self-reported dietary information obtained from food frequency questionnaires is subject to measurement error that could be attributed to recall bias and social desirability. Dietary SPD intake may not entirely reflect the actual SPD concentration in the body. The present study examines SPD levels in serum to objectively reflect the concentration of SPD in the body and provide a more objective basis for subsequent promotion and application.

Therefore, we explore the association between serum SPD levels and MCI based on the mutual verification of cross-sectional and longitudinal studies conducted on a Chinese population.

## Materials and methods

### Study population

We derived the data from a large-scale longitudinal study. The baseline survey was conducted from June 2019 to August 2019 in the rural areas of Fuxin County, Liaoning Province, China. Based on the demographic characteristics, two townships were selected from the southern region and one township was chosen from the northern and eastern regions of the county. A total of 33 villages were selected from these four townships. The inclusion criteria for participants included: (1) ≥35 years old; (2) a local residence time ≥5 years; and (3) signed informed consent. The exclusion criteria included: (1) pregnancy; (2) severe liver and renal failure; and (3) unwillingness to participate in this study. A total of 4,689 participants were recruited for the study. The follow-up survey was conducted from June 2021 to August 2021 with the same inclusion and exclusion criteria at baseline. In conclusion, 2,046 participants were enrolled as the longitudinal study population. Written informed consent was obtained from all participants. In the case of illiterate participants, we obtained written informed consents from their proxies. The procedures followed were performed under the ethical standards of the responsible committee on human experimentation of China Medical University ([2018]083).

**FIGURE 1:**
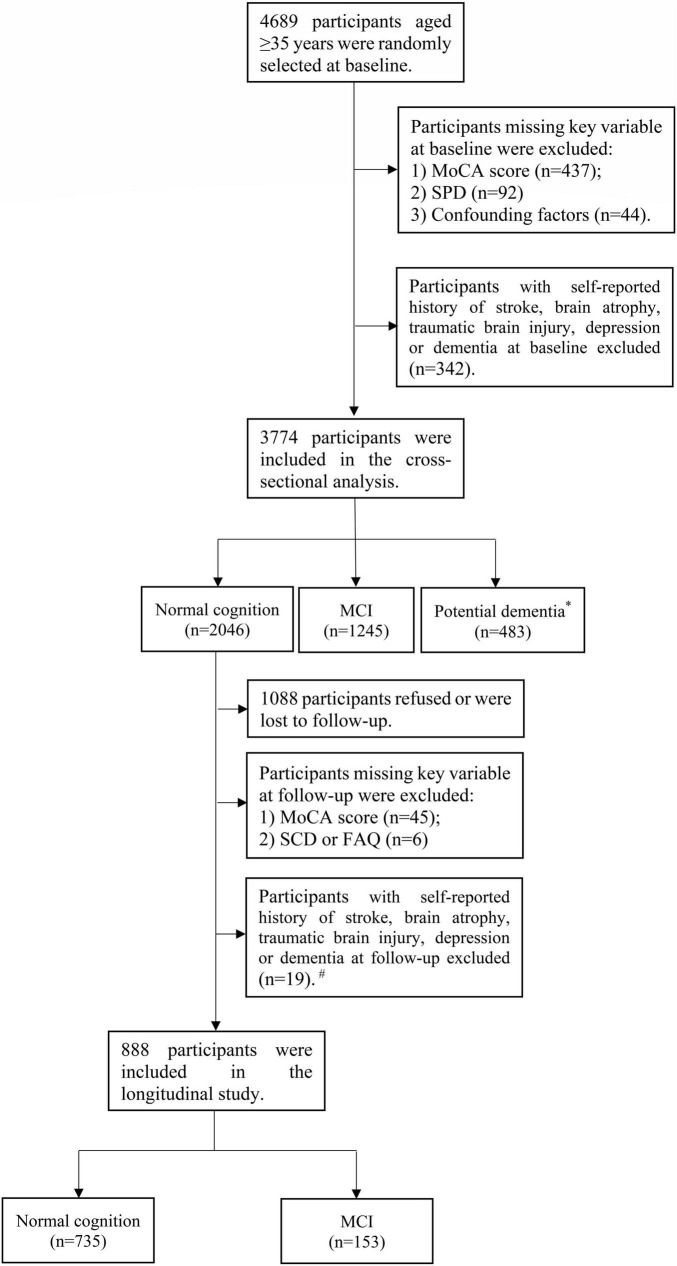
Flowchart of the participants included in the current analysis. *Potential dementia was defined following MoCA-BC. ^#^Dementia was diagnosed following the criteria of the NIA-AA workgroup.

Figure 1 shows a flowchart of the participants included in the current analysis. Of the 4,689 participants, those with missing information about Montreal cognitive assessment (MoCA) score (*n* = 437), SPD (*n* = 92), and confounding factors (*n* = 44) at baseline were excluded. Additionally, 342 participants with a history of stroke, brain atrophy, traumatic brain injury, depression, or dementia at baseline were excluded. At baseline, dementia was defined as a self-reported dementia diagnosis by a physician. In conclusion, 3,774 participants were enrolled in the cross-sectional analysis. For the longitudinal study, 2,046 participants with NC were declared eligible to attend the follow-up. Among these participants, 1,088 participants refused or were lost to follow-up. We further excluded participants with missing Montreal cognitive assessment-basic for Chinese (MoCA-BC) scores (*n* = 45) and subjective cognitive decline (SCD), or functional activities questionnaire (FAQ) (*n* = 6) at follow-up. Participants with a history of stroke, brain atrophy, traumatic brain injury, depression or dementia were also excluded at follow-up (*n* = 19). A total of 888 participants were enrolled in the longitudinal study.

### Measurement of serum spermidine

The participants were examined after an overnight fast. Blood samples were drawn from the antecubital vein in the morning and were collected into siliconized vacuum glass tubes. Serum was obtained by centrifugation at 3,000 rpm for 10 min and then stored at −80°C until further analysis. SPD levels in serum were measured using high-performance liquid chromatography with a fluorescence detector (HPLC-FLD). The main process of detecting serum SPD was as follows: SPD trihydrochloride was performed by adding 200 μL 0.1 M HCl to 100 μL serum. Protein precipitation was further conducted by adding 1 mL acetonitrile (ACN) to the mixture to precipitate the serum proteins. The supernatant was blown to dryness, following which 200 μL of 0.1 M HCl was added to reconstitute the dried product. Derivatization was performed by adding 200 μL dansyl chloride and 400 μL buffer to the above 200 μL reconstituted product. This was followed by vortexing, incubation in a water bath at 60°C for 45 min, cooling to room temperature, adding 40 μL ammonia water, incubation in the dark for 30 min, and making up the volume to 1 mL using ACN. This final solution was mixed well and passed through a 0.22 μm filter. The extracted samples were analyzed using an HPLC system to obtain a standard curve. The mobile phase solutions labeled A and B were ultrapure water and ACN, respectively. Gradient elution was performed as stated: 0–7 min: 55–50% A, 7–25 min: 50–10% A, 25–31 min: 10% A, 31–35 min: 10–55% A, and 35–40 min: 55% A. The flow rate was maintained at 0.8 mL/min and the column temperature was set to 35°C, while the detection wavelength was λ_*ex*_/λ_*em*_ = 340/510 nm.

### Mild cognitive impairment assessment

The Chinese version of the MoCA-BC is routinely used to screen the MCI of older Chinese adults with different education levels and this is a 30-point test covering nine cognitive domains. Supplementary Table 1 shows details on the components and corresponding maximum scores for each domain. At baseline, we only used MOCA-BC to screen for MCI and potential dementia. The optimal MoCA-BC cutoff scores used for MCI assessment were 19 for individuals with six or fewer years of education, 22 for those with 7–12 years of education, and 24 for those with more than 12 years of education (Chen et al., 2016). Participants with MoCA-BC scores <13 for individuals with six or fewer years of education, <15 for those with 7–12 years of education, and <16 for those with more than 12 years of education were potentially considered to have dementia (Huang et al., 2018).

At follow-up, the diagnosis of MCI was based on the criteria proposed by the National Institute on Aging-Alzheimer’s association (NIA-AA) workgroup (Albert et al., 2011). The criteria included the following: (1) cognitive complaints from participants confirmed by an informant (relatives/doctors), which were assessed using the SCD scale; (2) objective evidence of impairment in one or more cognitive domains, which were assessed using the MoCA-BC in this study; and (3) independence of daily functional ability, which was measured using FAQ. A score of FAQ ≥5 was defined as dysfunction consistent with dementia divided from NC; and (4) no dementia, based on the fifth edition of the diagnostic and statistical manual of mental disorders (DSM-V) (Sachdev et al., 2014).

### Assessment and definition of other variables

Relevant data on demographic variables (age, sex, ethnicity, and education level), lifestyle factors (smoking and alcohol consumption), and history of disease were collected using a standardized questionnaire. Smokers were defined as people who smoked at least one cigarette per day and continued to smoke for a minimum of 6 months, while drinkers were defined as people who take at least three drinks every week for a minimum of 6 months.

Height and weight were measured using standardized procedures. Body mass index (BMI) was calculated in terms of weight (kg)/height (m)^2^. According to the American Heart Association protocol, blood pressure (BP) was measured three times for at least 1 min after a minimum rest of 5 min between each measurement using a standardized automatic electronic BP measuring instrument (HEM-8102A/K) (O’Brien et al., 1990). The participants were instructed to avoid alcohol consumption, cigarette smoking, coffee/tea, and exercise for at least 30 min before the BP measurement. The average of three BP values was used for the final analysis and evaluation. Hypertension was taken as an antihypertensive medication in the last 2 weeks, diastolic blood pressure (DBP) ≥90 mmHg or systolic blood pressure (SBP) ≥140 mmHg (Joint Committee for Guideline Revision, 2019). Fasting blood samples were collected in the morning from participants who had fasted for at least 8 h. Fasting serum glucose was measured using a hexokinase method using the Roche Cobas 8000 C701 automatic biochemical analyzer in an accredited central laboratory. All the laboratory equipment was calibrated, and the blood samples of all the populations were randomly coded and tested blindly to effectively eliminate any systematic error, bias, or variability of laboratory batch measurements. Diabetes mellitus was defined as a condition in which fasting serum glucose was ≥7.0 mmol/L, using hypoglycemic drugs or insulin, or self-report of diabetes diagnosis by a physician or other health professional (Wang et al., 2021).

### Statistical analysis

Normally distributed data are expressed as the mean ± SD; SPD levels with a skewed distribution are reported as medians (interquartiles) and ln transformed to ensure approximate normality before analysis. Categorical variables are represented using frequency and percentage. Characteristics were compared using the one-way analysis of variance, non-parametric analysis or χ^2^ test, as appropriate. Multivariate logistic regression models were used to calculate the odds ratios (ORs) with 95% confidence interval (CI) for the relationships between SPD and MCI or cognitive domain dysfunction, adjusting for age, sex, ethnicity, education levels, smoking, drinking, BMI, history of hypertension, diabetes, and coronary heart disease (CHD). SPD entered the model as a tertile (T1: <16.69 ng/mL, T2: 16.69–37.29 ng/mL, T3: ≥37.29 ng/mL), with the lowest tertile (T1) as the reference group. We also used restricted cubic splines with three knots at the 25th, 50th, and 75th centiles to flexibly model the relationship of SPD with MCI. Additionally, receiver operating characteristic (ROC) curves were constructed, and the areas under the curves (AUCs) were calculated to assess the discriminant power of SPD for MCI. We further examined whether adding Ln (SPD) could improve the net reclassification index (NRI) and/or integrated discrimination improvement (IDI) for MCI. All analyses were conducted using SPSS 22.0 (IBM SPSS Inc., Chicago, IL, United States) and SAS statistical software (version 9.4, SAS Institute Inc., Cary, NC, United States).

## Results

**FIGURE 2:**
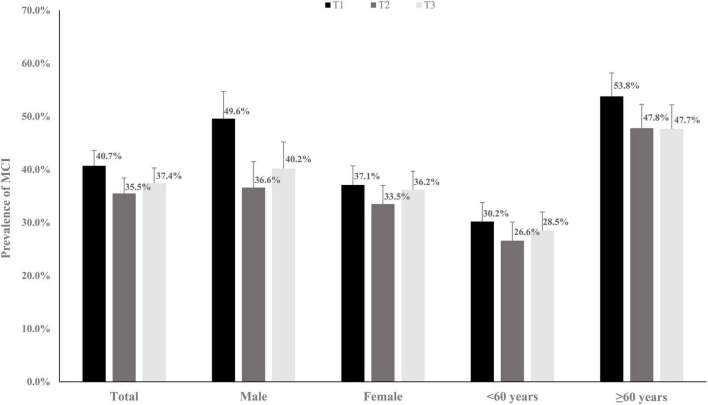
The prevalence rates (95% CI) of mild cognitive impairment (MCI) grouping by different spermidine (SPD) levels.

Table 1 shows the baseline characteristics of the 3,291 participants stratified by the SPD tertile. In total, the mean (SD) age was 57.4 (9.8) years. Sex, alcohol consumption, smoking, SBP, DBP, and BMI at baseline significantly differed among the three groups. Figure 2 shows the prevalence (95% CI) of MCI at baseline. The prevalence (95% CIs) of MCI for participants in the first, second, and third tertile groups was 40.7% (37.8, 43.6%), 35.5% (32.6, 38.8%), and 37.4% (34.5, 40.2%), respectively.

**TABLE 1:** Baseline characteristics stratified by spermidine (SPD) tertile.^a^

Characteristic	Total	T1 (<16.69 ng/ml)	T2 (16.69–37.29 ng/ml)	T3 (≥37.29 ng/ml)	*P*
Female, No. (%)	2,178 (66.2)	769 (70.3)	766 (69.3)	643 (58.9)	<0.001
Age, y	57.4 ± 9.8	57.2 ± 9.3	57.2 ± 9.9	58.0 ± 10.1	0.057
Ethnicity, No. (%)					0.367
Han ethnicity	2,134 (64.8)	685 (62.6)	726 (65.7)	723 (66.2)	
Mongolian	1,012 (30.8)	362 (33.1)	330 (29.9)	320 (29.3)	
Others	145 (4.4)	47 (4.3)	49 (4.4)	49 (4.5)	
Education level, No. (%)					0.805
Illiterate or primary school	1,279 (38.9)	417 (38.1)	431 (39.0)	431 (39.5)	
Junior high school	1,507 (45.8)	498 (45.5)	513 (46.4)	496 (45.4)	
Tertiary high school or higher	505 (15.3)	179 (16.4)	161 (14.6)	165 (15.1)	
Drinker, No. (%)	951 (28.9)	297 (27.1)	295 (26.7)	359 (32.9)	0.002
Smoker, No. (%)	1,129 (34.3)	358 (32.7)	351 (31.8)	420 (38.5)	0.002
History of CHD, No. (%)	432 (13.1)	145 (13.3)	131 (11.9)	156 (14.3)	0.238
Antihypertensive medicine use, No. (%)	226 (6.9)	175 (16.0)	180 (16.3)	157 (14.4)	0.413
Hypoglycemic medicine or insulin, No. (%)	512 (15.6)	82 (7.5)	78 (7.1)	66 (6.0)	0.388
BMI, kg/m^2^	24.8 ± 3.7	24.7 ± 3.8	24.5 ± 3.6	25.2 ± 3.8	<0.001
SBP, mmHg	132.6 ± 20.8	132.1 ± 20.8	131.5 ± 20.3	134.3 ± 21.2	0.003
DBP, mmHg	80.5 ± 11.1	80.0 ± 10.7	80.2 ± 10.9	81.3 ± 11.7	0.008
Fasting glucose, mmol/L	5.9 ± 1.7	5.9 ± 2.0	5.8 ± 1.5	5.8 ± 1.6	0.096
SPD, median (IQR), ng/mL	24.84 (13.41–49.28)	10.81 (9.13–13.39)	24.84 (20.89–30.77)	65.01 (49.28–95.99)	<0.001

SPD, spermidine; BMI, body mass index (calculated as weight in kilograms divided by height in meters squared); SBP, systolic blood pressure; DBP, diastolic blood pressure; CHD, coronary heart disease; SD, standard deviation; IQR, interquartile range.

^a^Unless otherwise indicated, data are expressed as the mean ± SD.

In cross-sectional analyses, high concentrations of serum SPD were associated with low MCI prevalence (Table 2). After adjusting for age, sex, ethnicity, education levels, smoking, drinking, BMI, history of hypertension, diabetes, and CHD, compared with the first tertile, the multivariate-adjusted ORs (95% CIs) for the second and third tertiles were 0.78 (0.65, 0.93) and 0.80 (0.67, 0.96), respectively.

**TABLE 2:** Association between serum SPD levels and prevalent MCI.*^a^*

	T1	T2	T3	*P*-value for non-linear trend
**Total (*N* = 3,291), OR (95% CI)**				
Events	445	392	408	
Model 1	1.00 (Ref.)	0.78 (0.66, 0.94)	0.80 (0.67, 0.95)	<0.001
Model 2	1.00 (Ref.)	0.78 (0.65, 0.93)	0.80 (0.67, 0.96)	<0.001
**Male (*N* = 1,113), OR (95% CI)**				
Events	187	133	150	
Model 1	1.00 (Ref.)	0.56 (0.42, 0.76)	0.63 (0.47, 0.85)	<0.001
Model 2	1.00 (Ref.)	0.56 (0.41, 0.76)	0.64 (0.47, 0.87)	< 0.001
**Female (*N* = 2,178), OR (95% CI)**				
Events	269	243	263	
Model 1	1.00 (Ref.)	0.86 (0.69, 1.07)	0.94 (0.75, 1.17)	0.107
Model 2	1.00 (Ref.)	0.85 (0.68, 1.06)	0.93 (0.75, 1.17)	0.108
**<60 years (*N* = 1,845), OR (95% CI)**				
Events	186	163	176	
Model 1	1.00 (Ref.)	0.86 (0.66, 1.10)	0.93 (0.72, 1.20)	0.045
Model 2	1.00 (Ref.)	0.86 (0.67, 1.12)	0.93 (0.72, 1.20)	0.045
**≥60 years (*N* = 1,446), OR (95% CI)**				
Events	260	230	230	
Model 1	1.00 (Ref.)	0.77 (0.60, 1.00)	0.74 (0.58, 0.96)	0.004
Model 2	1.00 (Ref.)	0.76 (0.58, 0.98)	0.76 (0.59, 0.98)	0.004

N, number of participants; SPD, spermidine; OR, odds ratio; CI, confidence interval; MCI, mild cognition impairment.

^a^ORs and CIs were calculated using logistic regression models. Model 1 was adjusted for age and gender. Model 2 was adjusted for age, gender, ethnicity, education levels, smoking, drinking, body mass index, history of hypertension, diabetes, and coronary heart disease.

In the subgroup analysis, we only observed an relationship between SPD and MCI among males and the subgrouping of participants into under 60 and over 60 years. After adjusting for age, ethnicity, education levels, smoking, drinking, BMI, history of hypertension, diabetes, and CHD in males, compared with the first tertile, the multivariate-adjusted ORs (95% CIs) for the second and third tertiles were 0.56 (0.41, 0.76) and 0.64 (0.47, 0.87), respectively. In people aged <60, after adjusting for confounding factors, compared with the first tertile, the multivariate-adjusted ORs (95% CIs) for the second and third tertiles were 0.86 (0.67, 1.12) and 0.93 (0.72, 1.20), respectively. In people aged >60, after adjusting for confounding factors, compared with the first tertile, the multivariate-adjusted ORs (95% CIs) for the second and third tertiles were 0.76 (0.58, 0.98) and 0.76 (0.59, 0.98), respectively.

**FIGURE 3:**
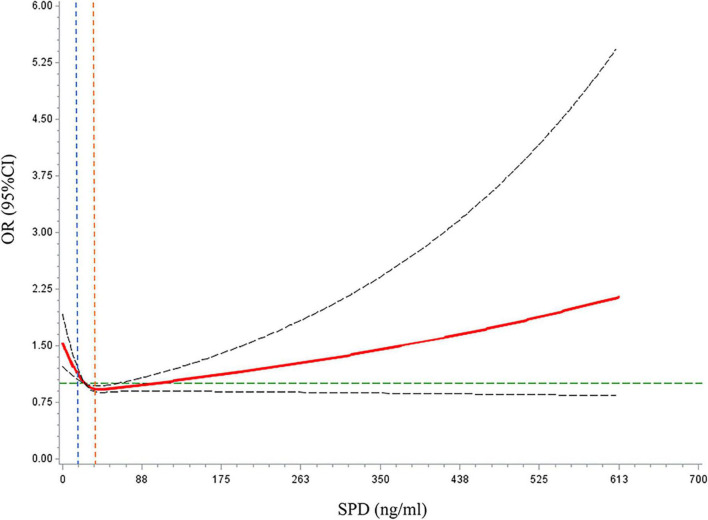
Dose–response relationships between spermidine (SPD) and mild cognitive impairment (MCI) prevalence. The solid red line represents the dose–response curve. The dashed black lines represented the 95% confidence interval. The dashed green line represents the reference line for OR = 1. The blue dashed line represents the reference line for the first tertile of SPD (SPD = 16.69 ng/ml). The orange dashed line represents the reference line for the third tertile of SPD (SPD = 37.29 ng/ml).

Restricted cubic spline models were used to evaluate the relationship between SPD and MCI (Figure 3). In multi-adjusted models, the relationships between SPD and MCI were non-linear (*P* for non-linear <0.001).

The ROC curves for the risk of MCI prevalence from multivariable logistic regression models, with and without SPD, were analyzed. The end points were characterized by MCI; therefore, the first model to incorporate age, sex, ethnicity, education level, smoking, drinking, BMI, history of hypertension, diabetes, and CHD had an AUC of 0.666 (95% CI: 0.648–0.685). Ln (SPD) was supplemented to Model 2 with an AUC of 0.668 (95% CI: 0.649–0.687). The difference in AUC was statistically insignificant, suggesting that the discriminative abilities of the two models were similar. Adding Ln (SPD) improved risk reclassification for MCI events, measured by continuous NRI (0.079; 95% CI, 0.009–0.149; *P* = 0.016) and by IDI (0.002; 95% CI, 0.000–0.003; *P* = 0.028). The NRI and IDI results showed that the power of the reclassification of the new model was significantly improved.

Additionally, we explored the relationship between SPD and different cognitive domains (Supplementary Table 2). In total, compared with the first tertile, the third tertile group had a decreased risk of fluency, orientation, calculation, abstract, and visual perception dysfunction, with multivariate-adjusted ORs (95% CIs) of 0.78 (0.63, 0.97), 0.78 (0.65, 0.94), 0.80 (0.67, 0.96), 0.79 (0.65, 0.96), and 0.76 (0.59, 0.96), respectively.

To further validate the above results, we analyzed the relationship between serum SPD levels and MCI prevalence using a longitudinal study (Table 3). After adjusting for age, sex, ethnicity, education levels, smoking, drinking, BMI, history of hypertension, CHD, and diabetes, and baseline MoCA-BC score, compared with the first tertile, ORs (95% CIs) for the second and third tertiles were 0.76 (0.49, 1.18) and 0.62 (0.39, 0.99), respectively.

**TABLE 3:** Association between serum SPD levels and incident MCI.*^a^*

	T1	T2	T3	*P*-value for non-linear trend
**Total (*N* = 888), OR (95% CI)**				
Events	62	49	42	
Model 1	1.00 (Ref.)	0.78 (0.51, 1.20)	0.61 (0.39, 0.95)	0.256
Model 2	1.00 (Ref.)	0.76 (0.49, 1.18)	0.62 (0.39, 0.99)	0.318

N, number of participants; SPD, spermidine; OR, odds ratio; CI, confidence interval; MCI, mild cognition impairment.

^a^ORs and CIs were calculated using logistic regression models. Model 1 was adjusted for age and gender. Model 2 was adjusted for age, gender, ethnicity, education levels, smoking, drinking, body mass index, history of hypertension, diabetes, coronary heart disease, and baseline MoCA-BC score.

## Discussion

We explored the role of serum SPD levels in MCI among 3,774 participants based on a large-scale epidemiological survey. We found a non-linear correlation between serum SPD levels and prevalent MCI events, which also showed potential of SPD to be a clinically useful indicator of MCI by improving risk reclassification. Similarly, in the longitudinal study, higher baseline SPD levels may indicate a lower risk of MCI events. To the best of our knowledge, this is the first study revealing the association of serum SPD levels with MCI in a large-scale population.

The relationship between SPD and cognition has been proven in animal experiments (Gupta et al., 2013; Hofer et al., 2021; Schroeder et al., 2021). Hofer et al. (2021) found that SPD is involved in preserving mitochondria and cognitive function. In aged fruit flies, simple SPD feeding suppressed age-induced memory impairment (Gupta et al., 2013). Most previous population studies on SPD and cognition examine dietary SPD (Wirth et al., 2018; Schwarz et al., 2020; Schroeder et al., 2021). A study involving 56 participants having cognitive declines and 47 healthy control participants showed that higher SPD intake might be a promising dietary approach to preserve brain health among older adults (Schwarz et al., 2020). The Bruneck Study shows that higher nutritional SPD intake is related to enhanced cognition in humans (Schroeder et al., 2021). A randomized controlled trial involving 30 participants with SCD showed that nutritional SPD was associated with a positive effect on memory performance (Wirth et al., 2018). Contrastingly, in another 12-month randomized clinical trial, long-term SPD supplementation in participants with SCD did not modify memory performance and biomarkers compared with placebo (Wirth et al., 2019). Additionally, Joaquim et al. (2019) monitored their study participants for 4 years and found that SPD levels were lower in both patient groups Alzheimer’s disease (AD; MCI) than in healthy controls. Graham et al. (2015) showed, through untargeted metabolomic analysis of human plasma, that polyamine metabolism was disrupted in patients with MCI.

Our results confirm that higher baseline serum SPD levels are associated with lower MCI prevalence and incidence, especially in males, and that subgrouping of participants into under 60 and over 60 years. The possible mechanism underpinning this association could be that SPD induces autophagy and exerts additional and potentially autophagy-independent metabolic and transcriptional effects *in vivo* (Madeo et al., 2018). However, cross-sectional analyses revealed a non-linear relationship between SPD and the risk of MCI, i.e., at very high SPD levels, the risk of MCI is elevated. We speculate that the elevated serum SPD levels in the MCI population are a compensatory effect in a pathological state. The geroprotective effects of SPD influence lifespan extension in a hormetic dose-dependent manner (Calabrese et al., 2020). In this hormesis scenario, a high dose of natural compounds is detrimental, because it inhibits antioxidant stress response pathways. The longitudinal study did not reveal a significant non-linear relationship of SPD with MCI. This finding is attributable to the small sample size (*N* = 888) of the longitudinal study and the high failure rate in conducting follow-up. There may be a certain loss to follow-up bias, thereby emphasizing the need for further examination using larger sample sizes.

Compared with previous studies, this study has several strengths. First, unlike previous clinical studies that included dozens of patients, we explore the relationship between serum SPD levels and MCI in a relatively large sample size (*N* = 3,774) of the general population for the first time. Second, there are three main sources of SPD in humans: cellular metabolism; oral absorption from dietary sources; or commensal gut bacteria (Madeo et al., 2018). Instead of assessing dietary SPD intake alone, we assessed overall SPD levels in the body to account for all SPD sources. Simultaneously, the measurement of SPD in serum can avoid the recall bias caused by the dietary assessment scale.

This study, however, also has inevitable limitations. First, we lacked an assessment of dietary SPD intake, which may affect the *in vivo* SPD level. Therefore, we could not establish a correspondence between serum SPD levels and dietary SPD intake. Second, we only measured the level of SPD once. Dynamically observing the changes in SPD in the blood may help to better identify its relationship with MCI over a time-course. Third, we exclusively examined the Chinese rural population, which may limit generalizability. The dietary culture of different populations differs and SPD is affected by diet (Madeo et al., 2020) and intestinal flora (Ramos-Molina et al., 2019). Therefore, the verification of the diverse population is meaningful.

## Data availability statement

The original contributions presented in this study are included in the article/Supplementary material, further inquiries can be directed to the corresponding authors.

## Ethics statement

The studies involving human participants were reviewed and approved by the procedures followed under the ethical standards of the responsible committee on human experimentation of China Medical University ([2018]083). The patients/participants provided their written informed consent to participate in this study.

## Author contributions

LZ and ZS designed the research. JX wrote the manuscript. QZ and FX provided essential reagents or provided essential materials. RZ, RL, and ZY conducted the research. YM performed statistical analysis. LZ was responsible for formulating all the sections of the manuscript. All authors contributed to the article and approved the submitted version.

## Conflict of interest

The authors declare that the research was conducted in the absence of any commercial or financial relationships that could be construed as a potential conflict of interest.

## Publisher’s note

All claims expressed in this article are solely those of the authors and do not necessarily represent those of their affiliated organizations, or those of the publisher, the editors and the reviewers. Any product that may be evaluated in this article, or claim that may be made by its manufacturer, is not guaranteed or endorsed by the publisher.

## Abbreviations

SPD: spermidine
SD: standard deviation
MCI: mild cognitive impairment
ORs: odds ratios
CI: confidence interval
MoCA: Montreal cognitive assessment
MoCA-BC: Montreal cognitive assessment-basic for Chinese
SCD: subjective cognitive decline
FAQ: functional activities questionnaire
HPLC: high-performance liquid chromatography
FLD: fluorescence detector
ACN: acetonitrile
NIA-AA: National Institute on Aging-Alzheimer’s association
DSM-V: the fifth edition of the diagnostic and statistical manual of mental disorders
NC: normal cognition
BMI: body mass index
BP: blood pressure
SBP: systolic blood pressure
DBP: diastolic blood pressure
CHD: coronary heart disease
ROC: receiver operating characteristic curves
AUC: areas under the curves
NRI: net reclassification index
IDI: integrated discrimination improvement.
