# Supplementary material for: Non-linear association between serum spermidine and mild cognitive impairment: Results from a cross-sectional and longitudinal study
Source: Front Aging Neurosci. 2022 Aug 2;14:924984. doi: 10.3389/fnagi.2022.924984 (PMC9380894; doi:10.3389/fnagi.2022.924984)
Supplement: Supplementary file 1 [file Table_1.DOCX]

Supplementary Material

# Supplementary Tables

| **Supplementary Table 1.** Cognitive domains assessed by MoCA. | |
| --- | --- |
| Items scores | Cutoffs of dysfunction |
| Executive function | ≤0/1 |
| Fluency | ≤1/2 |
| Orientation | ≤5/6 |
| Calculation | ≤2/3 |
| Abstract | ≤2/3 |
| Delay recall | ≤4/5 |
| Visual perception | ≤2/3 |
| Naming | ≤3/4 |
| Attention | ≤2/3 |

| **Supplementary Table 2.** Association between serum SPD levels and cognition domain at baseline. ^a^ | | | |
| --- | --- | --- | --- |
|  | T1 | T2 | T3 |
| Total, OR (95% CI) | | | |
| Executive function | 1.00 (ref.) | 0.85 (0.69, 1.04) | 0.82 (0.67, 1.00) |
| Fluency | 1.00 (ref.) | 0.93 (0.75, 1.15) | 0.78 (0.63, 0.97) |
| Orientation | 1.00 (ref.) | 0.91 (0.77, 1.09) | 0.78 (0.65, 0.94) |
| Calculation | 1.00 (ref.) | 0.84 (0.70, 1.01) | 0.80 (0.67, 0.96) |
| Abstract | 1.00 (ref.) | 0.99 (0.82, 1.20) | 0.79 (0.65, 0.96) |
| Delay recall | 1.00 (ref.) | 0.85 (0.69, 1.05) | 0.88 (0.71, 1.10) |
| Visual perception | 1.00 (ref.) | 0.69 (0.55, 0.88) | 0.76 (0.59, 0.96) |
| Naming | 1.00 (ref.) | 0.88 (0.72, 1.08) | 0.90 (0.73, 1.10) |
| Attention | 1.00 (ref.) | 0.97 (0.77, 1.21) | 0.98 (0.78, 1.24) |
| Male, OR (95% CI) | | | |
| Executive function | 1.00 (ref.) | 0.70 (0.50, 0.98) | 0.81 (0.57, 1.14) |
| Fluency | 1.00 (ref.) | 0.83 (0.54, 1.27) | 0.56 (0.37, 0.84) |
| Orientation | 1.00 (ref.) | 0.96 (0.71, 1.30) | 0.70 (0.52, 0.96) |
| Calculation | 1.00 (ref.) | 0.76 (0.56, 1.04) | 0.83 (0.61, 1.13) |
| Abstract | 1.00 (ref.) | 0.69 (0.50, 0.95) | 0.67 (0.48, 0.92) |
| Delay recall | 1.00 (ref.) | 0.60 (0.39, 0.92) | 0.73 (0.47, 1.13) |
| Visual perception | 1.00 (ref.) | 0.74 (0.49, 1.10) | 0.89 (0.59, 1.35) |
| Naming | 1.00 (ref.) | 0.83 (0.57, 1.21) | 1.12 (0.78, 1.60) |
| Attention | 1.00 (ref.) | 1.00 (0.67, 1.48) | 0.99 (0.67, 1.47) |
| Female, OR (95% CI) | | | |
| Executive function | 1.00 (ref.) | 0.99 (0.77, 1.28) | 0.87 (0.68, 1.12) |
| Fluency | 1.00 (ref.) | 0.90 (0.70, 1.15) | 0.96 (0.74, 1.23) |
| Orientation | 1.00 (ref.) | 0.87 (0.69, 1.08) | 0.89 (0.71, 1.11) |
| Calculation | 1.00 (ref.) | 0.80 (0.64, 1.00) | 0.84 (0.67, 1.06) |
| Abstract | 1.00 (ref.) | 1.10 (0.86, 1.39) | 0.92 (0.73, 1.17) |
| Delay recall | 1.00 (ref.) | 0.91 (0.71, 1.16) | 0.87 (0.68, 1.12) |
| Visual perception | 1.00 (ref.) | 0.67 (0.50, 0.89) | 0.75 (0.56, 1.01) |
| Naming | 1.00 (ref.) | 0.99 (0.77, 1.26) | 0.85 (0.66, 1.08) |
| Attention | 1.00 (ref.) | 0.96 (0.73, 1.26) | 0.98 (0.75, 1.29) |
| <60 years, OR (95% CI) | | | |
| Executive function | 1.00 (ref.) | 0.81 (0.63, 1.04) | 0.95 (0.74, 1.22) |
| Fluency | 1.00 (ref.) | 0.98 (0.75, 1.27) | 0.89 (0.69, 1.16) |
| Orientation | 1.00 (ref.) | 0.97 (0.77, 1.24) | 0.84 (0.66, 1.07) |
| Calculation | 1.00 (ref.) | 0.88 (0.69, 1.12) | 1.01 (0.80, 1.29) |
| Abstract | 1.00 (ref.) | 1.20 (0.94, 1.54) | 1.04 (0.81, 1.33) |
| Delay recall | 1.00 (ref.) | 0.93 (0.72, 1.21) | 0.82 (0.63, 1.07) |
| Visual perception | 1.00 (ref.) | 0.66 (0.50, 0.87) | 0.79 (0.60, 1.04) |
| Naming | 1.00 (ref.) | 1.17 (0.87, 1.58) | 1.08 (0.80, 1.46) |
| Attention | 1.00 (ref.) | 0.88 (0.62, 1.24) | 0.94 (0.66, 1.33) |
| ≥60 years, OR (95% CI) | | | |
| Executive function | 1.00 (ref.) | 1.00 (0.69, 1.44) | 0.74 (0.51, 1.05) |
| Fluency | 1.00 (ref.) | 0.80 (0.55, 1.17) | 0.67 (0.46, 0.97) |
| Orientation | 1.00 (ref.) | 0.85 (0.65, 1.10) | 0.74 (0.57, 0.97) |
| Calculation | 1.00 (ref.) | 0.77 (0.58, 1.03) | 0.64 (0.48, 0.86) |
| Abstract | 1.00 (ref.) | 0.79 (0.58, 1.08) | 0.58 (0.42, 0.79) |
| Delay recall | 1.00 (ref.) | 0.74 (0.51, 1.07) | 1.01 (0.68, 1.49) |
| Visual perception | 1.00 (ref.) | 0.84 (0.52, 1.37) | 0.75 (0.46, 1.21) |
| Naming | 1.00 (ref.) | 0.78 (0.59, 1.03) | 0.84 (0.63, 1.11) |
| Attention | 1.00 (ref.) | 0.99 (0.74, 1.34) | 1.12 (0.83, 1.51) |
| Abbreviations: SPD, spermidine | | | |
| ^a^Adjusted for age, gender, ethnicity, education levels, smoking, drinking, BMI and history of hypertension, diabetes and CHD. | | | |
